# A community-based single fall prevention exercise intervention for older adults (STEADY FEET): Study protocol for a randomised controlled trial

**DOI:** 10.1371/journal.pone.0276385

**Published:** 2022-10-20

**Authors:** Rebecca Hui Shan Ong, Milawaty Nurjono, Junisha Jumala, Raymond Choon Chye Teo, Gek Kheng Png, Poh Choo Tan, Mong Nee Kee, Hong Choon Oh, Moi Kim Wee, Karen Lai Ming Kan, Lina Farhana Binte Rosle, Christopher Tsung Chien Lien, Shou Lin Low

**Affiliations:** 1 Department of Health Services Research, Changi General Hospital, Singapore, Singapore; 2 Department of Rehabilitative Services, Changi General Hospital, Singapore, Singapore; 3 Singapore Sport & Exercise Medicine Centre, Changi General Hospital, Singapore, Singapore; 4 Chief Nurse, Changi General Hospital, Singapore, Singapore; 5 Department of Community Nursing, Changi General Hospital, Singapore, Singapore; 6 Department of Community and Mental Health, Changi General Hospital, Singapore, Singapore; 7 Department of Geriatric Medicine, Changi General Hospital, Singapore, Singapore; GERMANY

## Abstract

**Background:**

Falls and fall-related injuries in older adults are a leading cause of disability and death. Evidence has shown the benefits of exercises in improving functional outcomes and reducing fall rates among community-dwelling older adults. However, there is lack of effective community-based single exercise intervention for a broad population of older adults who are at high risk for falls. We aim to evaluate the effectiveness of Steady Feet (SF), a 6-month tailored community fall prevention exercise programme for improving functional outcomes. SF classes are facilitated by community fitness instructors and an exercise video. The main outcome is between-group changes in short physical performance battery (SPPB) scores. Secondary outcomes include balance confidence, fear of falling, quality of life, fall rates, and cost effectiveness.

**Methods:**

We present the design of a 6-month randomised controlled trial of 260 older adults (≥ 60 years old). Individuals will be randomised in a 1:1 allocation ratio to the SF group or usual care group. Participants will be assessed at baseline, 3-month, and 6-month. Data on socio-demographics, co-morbidities, balance confidence, fear of falling, quality of life, physical activity level, rate of perceived exertion, fall(s) history, healthcare utilisation and cost, and satisfaction levels will be collected. Participants will also undergo functional assessments such as SPPB. Moreover, providers’ satisfaction and feedback will be obtained at 3-month.

**Discussion:**

An effective community fall prevention programme may lead to improved functional outcomes and reduced fall rates. Findings will also help inform the implementation and scaling of SF nation-wide.

**Trial registration:**

**Clinicaltrials.gov registration:** NCT04801316. Registered on 15^th^ March 2021.

## Introduction

Falls and fall-related injuries in older adults are of public health concern [1, 2], as nearly one in three older adults fall at least once every year [3]. Falls can lead to adverse consequences like hospitalisation, fractures, poor psychological well-being, reduced mobility [4–7], and injury-related deaths [8–10]. Falls also exert significant economic burden on individuals and society [11, 12]. Singapore is a Southeast Asian multi-ethnic urban city state, and is projected to be the second-fastest aging population in the world between 2019 and 2050 [13]. About 18.6% (nearly 1 in 5) Singaporean older adults aged 60 years and above reported at least one fall during the past 12 months [14]. In 2009, falls accounted for 85% of all elderly trauma cases seen at the emergency departments (ED) across the nation [15]. This number is expected to rise with the rapidly aging population, hence, there is a pressing need to address the issues associated with falls among older adults in a timely manner.

Trials and systematic reviews have provided evidence that exercise interventions containing strength and balance components are effective in improving functional outcomes and reducing fall rates in older adults [4, 16–18]. Compared to usual care, multifactorial interventions (comprising of three or more intervention components), or single interventions such as exercises or risk assessments were found to be more effective in reducing fall rates [4, 19]. In terms of magnitude of effectiveness, single exercise interventions have been shown to be similarly or more effective than multifactorial interventions among community-dwelling older adults [16, 20–22]. Building on evidence, single intervention is also preferred in the community because multifactorial interventions are comparatively more costly and complex to implement [20, 21]. However, there is a gap in the evidence for an effective community-based single exercise intervention for a broad population of older adults at high risk for falls.

To address this, a 6-month fall prevention community group exercise programme “Steady Feet” (SF) was developed by Changi General Hospital (CGH), Singapore. SF is a single exercise intervention based on established exercises that are also embraced by the Steps to Avoid Falls in the Elderly (SAFE), and the Otago Exercise Programme (OEP) [17, 23]. Healthcare professionals and key community stakeholders such as senior activity centres (SAC), SportsSG, a national government agency for sports, and NTUC Health, a social enterprise with elderly-related services were engaged throughout the development and implementation process. The development of SF was planned to be conducted in 2 phases. Firstly, the proof-of-concept (POC) aimed to examine the feasibility and acceptability of SF. Secondly, the proof-of-value (POV), which is presented in the current paper, aims to examine the effectiveness of SF. The team recently completed the POC phase. The POC enabled us to pilot test the processes and study materials (e.g., questionnaires), allowing improvements and modifications to be made prior to the bigger POV effectiveness study. Our initial findings support feasibility and acceptability of SF among community dwelling adults in Eastern part of Singapore. This manuscript describes the assessment of SF effectiveness using a Randomised Controlled Trial (RCT) design according to the SPIRIT statement [24].

## Materials and methods

### Study design and hypothesis

Designed as an open-label, two arm, parallel group, RCT, 260 community-dwelling older adults (≥ 60 years old) at high risk for falls will be recruited and randomized into (1) exercise group (intervention), or (2) usual care group (control). All participants will attend a total of 3 study visits over a period of six months, at baseline, 3-, and 6-month after randomisation. Additionally, participants in the intervention group will take part in SF between baseline and 6-month. The primary outcome of interest is the difference in changes in Short Performance Physical Battery (SPPB) scores between the intervention and control groups from baseline to 6^th^ month. We hypothesised that the intervention group will show ≥ 0.6 points improvements in SPPB scores relative to the control group. In literature, a meaningful improvement in SPPB scores for older adults was described to be between 0.3 and 0.6 [25–27]. For secondary outcomes, we hypothesised that compared to the control group, the intervention group will show more improvements in balance confidence, fear of falling, quality of life, fall rates, and the SF programme will be more cost effective compared to usual care.

### Recruitment

The adapted Fall Risk for Older People – community setting (FROP-COM) screener [28] and the single leg stance (SLS) [29], have a moderate to high level of accuracy in identifying future falls among community-dwelling older adults [30, 31]. These instruments will be used to screen for falls risk during the first level screening (“Level 1”) by leveraging on CGH’s chronic illness community screening programmes or Community Nursing Posts (CNP). CNPs are located within senior care facilities, community partners sites (e.g., social service offices), faith-based organisations (e.g., churches; mosques) and community centres. Those identified to be at high risk for falls will be referred to a “Level 2” screening, which are held at the same locations as “Level 1” for comprehensive geriatric assessments, vision tests, and cognitive tests, and results are used to assess study eligibility. Participants who meet the following inclusion criteria at Level 2 screening will be eligible for the study:

1. Aged 60 years old or above.
2. SPPB score from 7 to 10
3. Pass at least 2 out of 3 Vision Function Test(s) (LogMar) vision, Stereoscopic vision, and Melbourne Edge Test (MET))
4. Does not possess significant cognitive impairment (AMT ≥ 5)

Participants will be excluded if they meet any of the following criteria:

1. Aged below 60 years old.
2. SPPB score of ≥ 11 or ≤ 6
3. Did not pass at least 2 out of 3 Vision Function Test(s) (LogMar vision, Stereoscopic vision, and MET)
4. Possess significant cognitive impairment (AMT < 5)

A study staff will introduce the study to eligible individuals and inform participants that they can choose to withdraw from the study at any point without penalty. Trained study staff will obtain written informed consent from all older adults prior to study participation.

### Allocation/Randomisation

A biostatistician who is independent from the study team will perform the randomization using a computer-generated random number sequence, with a 1:1 allocation ratio, and prepare a stack of sealed envelopes containing the allocated group. Allocation assignment is concealed from the study team. At the end of “Level 2”, a delegated study staff will open the sealed envelope, starting from the first envelope at the top of the stack and record the allocated group for each enrolled participant. Participants will be informed of their assigned groups by study staff. Study staff and participants are unblinded to study conditions.

### Study interventions

Participants assigned to the intervention group will be provided with CGH’s usual care for their conditions, which are fall prevention educational materials and advice, invited to participate in SF classes, and to attend all study visits. The 6-month SF programme is divided into two phases: (i) Steady Feet Intensive (SFI) and (ii) Steady Feet Maintenance (SFM). SFI is a structured, supervised, 1-hour intensive group exercise class, conducted either in-person or virtually (dependent on the prevailing national COVID-19 guidelines) on two non-consecutive days on a weekly basis, for 3 months (24 sessions). The SFI exercises were based on established exercises incorporating strength and balance components [17, 23], and focuses on strengthening the lower extremities, improving static balance, flexibility, and weight shifting. Steps were tailored to suit the local older adult population through the work of a local expert panel that comprised of CGH physiotherapists, exercise physiologist, and geriatricians.

To achieve sustainability, SF aims to develop and empower SportsSG’s community fitness instructors (FI) in implementing an effective falls prevention programme in a consistent manner in community-based settings. FIs have a background in exercise or sports and assist in the operations of SportsSG sports facilities. Prior to SF class commencement, FIs are required to undergo a train-the-trainer programme (TTT). The TTT is designed to grow a self-sustaining pool of FIs who can continue to deliver SF with high fidelity in community settings. It consists of a 5-hour CGH physiotherapist-led training session that covers class preparation, SFI exercise steps, administration of functional assessments and class engagement. FIs will be assessed with a competency checklist by the trainers, and those who did not meet the acceptable level will undergo additional training until they are deemed sufficiently competent. At least one FI is assigned to each SFI class and will conduct the class alongside an exercise video in accordance with the protocol. SFI classes will commence within two weeks from the baseline assessment. Class sizes range from 4 to 15 participants. SFI comprises of 10 sets of exercises (i.e., forward step knee lift, forward lunge, four steps forward and four steps backward, tandem walk, heel walk, toe walk, sideways walk, figure of 8 walk, butt kick, half squat). Participants are encouraged to repeat each set as many times as possible within 1 min, followed by a 30 second rest. Each class session will begin with a 5-10 min warm-up, followed by 35-45 min of exercises, and end with a 5-10 min cool down. The exercise video covers the warmup, SFI exercises, and cool down, three instructors in the video will concurrently demonstrate the same set of exercise in three different ways, either with mobility aids, the standard method, or with progression. The initial level of the exercise will be tailored according to each participant’s capability through discussions between FIs and participants. For example, the forward lunge can be performed with assistance from a walking cane (mobility aid) or a chair for those requiring additional support. FI(s) will monitor participants throughout the classes and recommend appropriate structured, gradual progression or regression of the exercise steps accordingly.

SFM consists of a 1-hour community group exercise class, held once weekly, for 3 months after the completion of SFI. A recommended list of SFM programmes will be provided to participants by the end of their last SFI class. This list will comprise of (i) SF exercise programme at a reduced frequency of one time per week and (ii) SportsSG or NTUC Health community group exercise programmes with balance and strength components, curated by the study’s physiotherapists and exercise physiologist. Participants will be asked to choose a programme from the list, after which, the whole class will proceed onto the exercise programme chosen by majority of participants in the class. If participants miss two or more consecutive SFI or SFM classes, study staff will contact them and attempt to re-engage them. SFI and SFM classes will be conducted at public community spaces (e.g., void decks, community centres), SAC premises, and SportsSG public sports facilities (e.g., ActiveSG gyms, dance studios) located in Eastern Singapore. These easily accessible venues were deliberately selected to promote uptake and continuation.

Participants assigned to the control group will be provided with CGH’s usual care for their conditions, which are falls prevention educational materials and advice, requested to maintain their lifestyle, and asked to attend all study visits. They will not undergo the SF programme. Additionally, phone calls will be made to all participants once every 1-2 months to check on them and answer any queries that they might have.

### Outcomes and data collection methods

**Fig 1:**
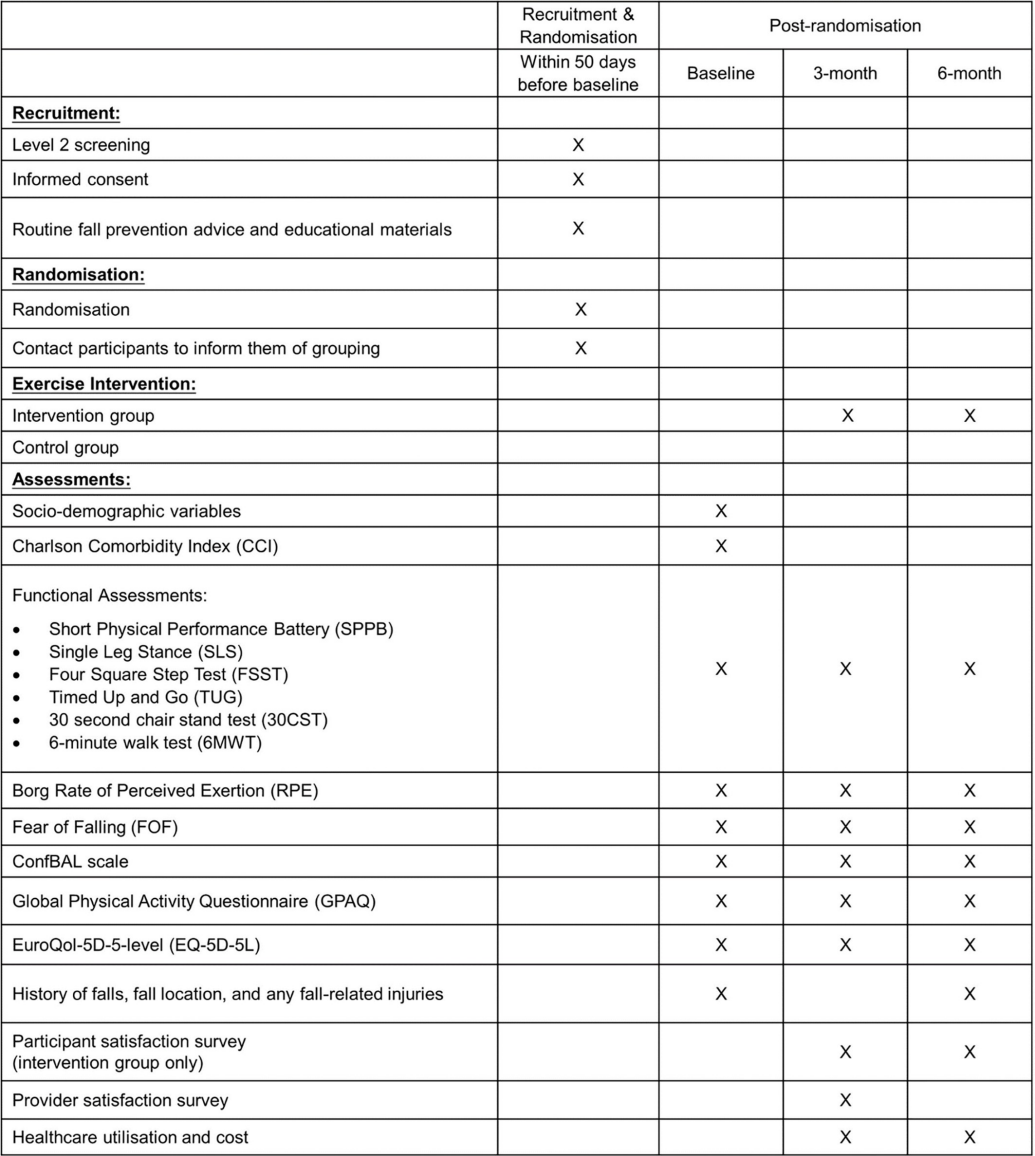
Steady Feet (SF) POV schedule of recruitment, interventions, and assessments.

Measurements for all participants will be collected at Day 1 (baseline), Day 90, and Day 180, with a 30-day grace window period to accommodate the planning and scheduling. SFI classes for individuals in the intervention group will commence within 2 weeks from baseline. All assessments and questionnaires will be administered by study staff during the study visits (Fig 1). At baseline, all participants’ socio-demographic information, and co-morbidities, will be collected. At each study visit, information on balance confidence, fear of falling, quality of life, physical activity level, rate of perceived exertion will be collected. Functional assessments such as short physical performance battery (SPPB), single leg stance, four square step test, timed up and go, 30 second chair stand test, 6-minute walk test will also be administered. A detailed falls history will be recorded at baseline, and 6-month. Healthcare utilisation and cost, and satisfaction levels of participants in the intervention group will be captured at 3-, and 6-month. Provider satisfaction survey will be administered to providers (e.g., fitness instructors, SF administrators, SAC staff and community nurses) at 3-month.

### Study measures

#### Primary variable

The SPPB is a well-validated and widely used tool that measures three components (i) balance (ii) gait speed and (iii) chair stands timing, which reflect lower body strength and endurance. An overall SPPB score will be calculated from these components and summed. Higher scores indicate better function whereas lower scores are noted to be associated with a higher risk of falls in older adults [32, 33]. For the balance tests, FIs will first demonstrate the positions, after which participants will be asked to stand unassisted in the order of these 3 positions: a) side-by-side stand; feet in a side-by-side position, b) semi-tandem stand; heel of one foot is beside the big toe of the other foot, and c) tandem stand; heel of one foot is in front and touching the toes of the other foot. Assistance can be provided by FIs to stabilise the participant before test commencement. Participants are asked to maintain each position for 10 seconds. If participants are unable to hold the position for 10 seconds, the balance tests will be stopped, and participants will proceed to the gait speed test. Participants will be scored according to Table 1.

**Table 1:** Scoring details for SPPB balance component.

Balance Tests	Scoring
Side-by-Side Stand	a. Held for 10 seconds = 1 point
	b. Not held for 10 seconds = 0 points
Semi-Tandem Stand	a. Held for 10 seconds = 1 point
	b. Not held for 10 seconds = 0 points
Tandem Stand	a. Held for 10 seconds = 2 points
	b. Between 3 and 9.99 seconds = 1 point
	c. Less than three seconds = 0 points

To measure gait speed, participants will be asked to walk a 4-metre course with their usual speed and are allowed to use assistive devices if needed. The best of two timings (in seconds) will be recorded. Scores will then be assigned to participants based on their timing: i) Unable to complete the walk in 60 seconds = 0 points, ii) < 60 seconds but > 8.70 seconds = 1 point, iii) 6.21 to 8.70 seconds = 2 points, iv) 4.82 to 6.20 seconds = 3 points, v) less than 4.82 seconds = 4 points. The chair stand test will be performed using a straight-backed chair, placed with its back against a wall. Participants are asked to stand up from the chair as quickly as possible without using their arms for 5 consecutive times. The time taken is recorded in seconds. Scores are assigned as such: i) Unable to complete or completes stands in more than 60 seconds = 0 points, ii) < 60 seconds but > 16.69 seconds = 1 point, iii) 13.70 to 16.69 seconds = 2 points, iv) 11.20 to 13.69 seconds = 3 points, v) less than 11.20 seconds = 4 points.

#### Secondary variables

The CONFbal scale is a self-reported 10 item measure of balance confidence [34]. Participants will be asked to rate their balance confidence on each item on a scale of 1 to 3 (not confident = 3, slightly confident = 2 and confident = 1). Ratings will be summed to form an overall CONFbal score (ranges from 10 to 30). Lower scores reflect fewer issues with balance confidence. The fear of falling (FoF) is a visual analogue scale ranging from 0 (not afraid) to 10 (extremely afraid) that captures a participant’s self-rated fear of falling.

The number of falls, defined as “an event which results in a person coming to rest inadvertently on the ground or floor or other lower level” [35], location(s) of the fall(s), and any fall-related injuries sustained within the last 6 months will be collected during the study visits based on participant’s self-reports.

Quality of life will be assessed using the EQ-5D-5L [36], a self-reported measure of health in five dimensions (i) mobility, (ii) self-care, (iii) usual activities, (iv) pain/discomfort, and (v) anxiety/depression, with five levels of severity. A health state utility score will be derived from the instrument. The number of healthcare utilisation episodes and its associated costs, reported in Singapore dollars (SGD) will be collected over the study period. These include cost associated with ED visits, Specialist Outpatient Clinic visits, primary care visits, and hospital admissions.

The 6-minute walk test (6MWT) is commonly used to evaluate aerobic capacity and endurance [37, 38]. It is performed along a 10-metre marked course. Participants will be asked to walk forth and back along the course, as far as possible, for 6 minutes, and the distance (metres) walked will be recorded.

SLS is a widely used and reliable measure of static balance in older adults [29, 39]. Participants will be asked to balance once on each leg without any assistance, and their timing (seconds) will be recorded.

Timed Up and Go (TUG) is used to examine balance, gait movements and dynamic turning mobility in community-dwelling older adults [40, 41]. Participants will be asked to rise from a chair, walk 3 metres forth and back, and return the seat. Their timing (in seconds) will be recorded.

Four Square Step Test (FSST) is a validated and reliable measure of dynamic standing balance in older adults [42]. Participants are asked to execute a pre-determined sequence by stepping through four squares demarcated on the ground. Their timing (seconds) will be recorded.

The 30 second chair stand test (30CST) is part of the Fullerton Functional Fitness Test (FFT) battery [43] and is commonly used to evaluate lower body strength in community-dwelling older adults [44]. Participants will be asked to rise from a chair with arms crossed, and to complete as many full stands as possible within 30 seconds. The number of full repetitions will be recorded.

#### Other variables

Baseline demographics and clinical indicators will be obtained from participants’ screening records. These include age, sex, ethnicity, marital status, education level, residential type, living arrangement, weight, height, and Charlson Comorbidity Index (CCI) scores. Study staff will also record exercise class attendances in study logs for participants in the intervention group over the study period.

The Global Physical Activity Questionnaire v2 (GPAQv2) will be used to capture participants’ physical activity level throughout the study, and to explore if participants continue to stay physically active after the intervention. It comprises of 16 items on sedentary behaviour, and physical activity levels in three domains 1) at work, 2) during travel, 3) recreational. The World Health Organisation’s GPAQv2 scoring protocol will be utilised to summarize the overall levels of physical activity, total MET values per week, and domain specific MET values (i.e., work, travel, recreation) [45].

Participants’ satisfaction of SF will be captured using a survey developed for the study. The survey will only be administered to the intervention group and consists of 31 questions covering these domains: (i) instructor, (ii) exercise steps, (iii) exercise venue, (iv) schedule, frequency, and duration of exercise classes, (v) effect of exercise class, and (vi) willingness to pay. Participants will be asked to rate their satisfaction on a five-point Likert scale (1= strongly disagree, 5 = strongly agree) and to provide feedback about SF. Follow-up surveys will capture whether participants took part in exercise programmes, details of these programmes, and reasons for exercise non-participation in exercises (if applicable). Providers’ satisfaction and feedback survey will be administered to individuals who were involved in the planning, developing and implementation of SF. It consists of 26 questions covering two domains: (i) implementation of screening and recruitment activities, and (ii) implementation of exercise classes. Providers will be asked to rate their satisfaction on a six-point Likert scale (1= strongly disagree, 2 = disagree, 3 = neutral, 4 = agree, 5 = strongly agree, and NA) and requested to provide any feedback about SF.

### Sample size

The primary outcome is the difference in mean SPPB scores between intervention and control group at 6-month. Assuming a 5% error margin, to have 80% statistical power to detect a 0.6 points difference in SPPB score changes with standard deviation of 1.5, 100 participants per group is required. Based on our pilot study, we estimate that 25% of participants will be lost to follow-up at 6-month. Hence, the study will aim to recruit a total sample of 260 participants. Sample size calculations were done with G*Power (ver. 3.1.9.4) [46]. There will be no replacement and no further data collection if a participant withdraws.

### Data analysis

All primary analyses will be conducted using the intention-to-treat principle (ITT). A secondary per protocol analysis (PP) will also be performed. The last value carried forward (LVCF) method will be used to input missing observations [47, 48]. A *p* < 0.05 will be considered as statistically significant. Statistical analysis will be performed using SPSS statistical software, version 26.0 (IBM Corp. Armonk, NY, USA). Categorical data will be presented as frequency (percentage), while continuous data will be presented as mean (standard deviation) for parametric distribution and median (interquartile range) for non-parametric distribution. Group comparisons of categorical will be performed using the chi-square test or Fisher’s Exact test, and the two-sample t test or Mann-Whitney U test will be utilized for continuous variables. If data transformations (e.g., logarithmic) are used to improve distribution characteristics (e.g., normality, variance homogeneity) for parametric procedures, they will be described in reporting of statistical results. Outcomes will be examined using a Condition (intervention vs. control) x Time (baseline v 6^th^ month outcomes) mixed model ANOVA. We will also conduct an exploratory analysis for Condition x Time (i.e., all time points), using a mixed model ANOVA. Rates will be calculated by dividing the number of events by time, and examined using Poisson or Negative Binomial regression, respectively. We will be adopting the health system perspective for the cost analysis. Results for cost–utility analysis will be presented as cost per quality-adjusted life years (QALY). To calculate QALY, responses to the EQ-5D-5L will be converted to a utility score using Singapore’s value set. To examine cost-effectiveness, we will calculate the incremental cost-effectiveness ratio (ICER) of the program as difference in costs between both groups divided by between-groups difference in QALY. We will adopt the World Health Organisation’s (WHO) suggestion that a per capital gross domestic product (GDP) threshold is sufficient to determine cost–effectiveness [49]. If the result was less than Singapore’s annual GDP, the intervention will be considered cost-effective.

### Study management and monitoring

The principal investigator (PI) will ensure that study staff are trained on the protocol, proper use of data collection form, and study assessments, before study initiation. The study team will conduct, at least, quarterly meeting to review the study data. Study data to be reviewed will include compilation of data obtained from study and any adverse events and/or serious adverse events recorded to ensure that patient safety is adhered. Research data will be stored in a password protected laptop within the research site. Screening forms, source documents, study logs, and signed informed consent forms will be stored in a locked cupboard in the research site office, and only accessible by the delegated study staff members.

#### Confidentiality of data and patient records

Participants will be assigned study code numbers. The data of all participants that are collected for study purposes will only be accessible to the PI and delegated study team members. The PI and delegated study staff will have access to the final trial dataset. The PI and CGH will permit study-related monitoring, audits and/or IRB review and regulatory inspection(s) to have direct access to source data/document.

#### Data monitoring

There is no data monitoring committee (DMC) for this study. The PI and co-investigators will regularly monitor the study to verify the accuracy and completeness of data, that the safety and rights of participants are protected, that the latest approved study protocol is being followed, and ensure that the conduct of the study is in accordance International Conference on Harmonisation Good Clinical Practice (ICH-GCP) and all applicable regulatory requirements. Any protocol deviations will be noted in the study records and reported in accordance with the ethics board requirements.

#### Harms

The study team will evaluate the wellbeing of the participants throughout the duration of the study. If the PI considers that continued participation could be harmful to an individual participant’s health or safety, then the participant will be withdrawn from the trial. If the findings have wider implications for all participants, then an investigation will be conducted to determine whether the trial should be continued or modified or terminated.

#### Recording and reporting of serious adverse events (SAEs) and adverse events (AE)

A serious adverse event (SAE) is any untoward medical occurrence that:

- Results in death.
- Is life-threatening (immediate risk of death).
- Requires inpatient hospitalization or prolongation of existing hospitalization.
- Results in persistent or significant disability/ incapacity.
- Results in congenital anomaly/birth defect.
- Is a medically important event.

All SAEs will be collected, documented, and assessed by the PI and delegated study team members. Only related SAE will be reported to the ethics board in accordance with its guideline. Related means there is a reasonable possibility that the event may have been caused by participation in the research. The PI is responsible for informing the ethics board after first knowledge that the case qualifies for reporting. Complaints and other adverse events will be recorded in the Study Site File. CGH will provide the appropriate medical treatment if the participant followed the instructions of the study team and is injured due to the study procedures.

#### Auditing

CGH’s appointed study monitor will schedule planned monitoring visits throughout the study to ensure that the study is conducted in accordance with the approved protocol, and the rights and safety of the participants are protected.

### Ethics and dissemination

#### Ethics approval

The trial is conducted in accordance with the ethical principles that have their origin in the Declaration of Helsinki and that are consistent with the Good Clinical Practice and the applicable regulatory requirements. The Centralised Institutional Review Board (CIRB) has approved the study protocol (CIRB Ref: 2020/3152). This study was registered at clinicaltrials.gov as NCT04801316.

#### Dissemination

The study results will be published in local and international journals. In addition, the results will be presented at conferences as well as to stakeholders and senior management.

## Discussion

In this paper, we present a protocol to determine the effectiveness of a single exercise intervention (Steady Feet) on community-dwelling older adults with high fall risk. The SF intervention combines strength and balance exercises to improve common modifiable risk factors for falls in older adults, including muscle weakness, balance deficits, and unstable gait [50, 51]. It builds on previous knowledge of best practiced fall prevention exercise programmes [17, 23]. The economic burden of falls is significant and is estimated at US$23.3 billion and US$1.6 billion per year in the United States, and the United Kingdom, respectively [11]. Faced with a rapidly aging population, Singapore is also expected to face a rising economic impact of falls. To our knowledge, only one study has reported on the cost-effectiveness of a multifactorial fall prevention programme in Singapore [52]. Hence, there are limited data in Singapore on how much cost utility community fall prevention programmes can provide. To the best of our knowledge, this is the first study in Singapore to examine the effectiveness and cost utility of a single exercise intervention among a broad population of older adults at high risk for falls. Insights gathered from this study is expected to address the current knowledge gaps and contribute to the literature to enable policy makers and healthcare professionals to make evidence-based decisions on falls prevention healthcare policies or interventions.

Sustainability has been identified as one of the common challenges faced when implementing community-based interventions. This has been previously attributed to reasons such as uncertainty about programme effectiveness, inadequately trained manpower, the lack of involvement and coordination from organizations or individuals trusted by the community of interest, and financial constraints [53–55]. To mitigate these, the SF programme plans to leverage on a capacity building model through partnerships in the community by investing resources in the training of FIs, and engaging key community partners in the early stages of programme development and implementation. Feedback from providers will also be collected during POV, as such inputs can lead to programme improvements, which could in turn fuel programme sustainability [56, 57]. Providers in this study represent a range of different perspectives, from screeners (for example, community nurses, and SAC staff), to exercise implementors (for example, fitness instructors) and administrators (for example, programme managers, heads of departments), who are important for identifying implementation gaps in a timely manner. This provides confidence in the relevance of study findings and the potential of recommendations being used for improvements.

In addition, the “gold standard” RCT design adopted provides great internal validity by minimising selection bias and confounding due to differences in population. It is also acknowledged that an RCT allows causation to be better established. Another strength of this study is the collection of a wide range of measurements. Such data will provide the study team with comprehensive insights required for making programme related refinements and decisions.

This study has some limitations. As recruitment and research sites are concentrated in the East, the current SF study population will be mainly limited to older adults in residing in the Eastern part of Singapore, potentially limiting the generalizability of the research findings. The study team acknowledges that participants included in this study may also be pursuing other physical activities while in the study which may potentially confound the study results. To mitigate such confounding effects, GPAQv2 will be administered during study visits to estimate and monitor participants’ physical activity levels and controlled for during analysis. Lastly, although in-person attendance during exercises is the default option, unanticipated COVID-19 pandemic restrictions might affect the study’s implementation through group size limitations, and reduction in usage of facilities. Hence, the team is exploring strategies for remote participation and planning for additional subgroup analyses to try to mitigate the impact, which may potentially make results harder to interpret.

Our protocol is timely as it addresses the need to developing an effective community-based single fall prevention exercise programmes for Singapore’s rapidly aging population, and will add valuable findings to the knowledge of cost utility of community fall prevention programmes [13]. The outcomes from this programme have important implications for older adults at risk for falls, and the data obtained will help inform the scaling and implementation of the Steady Feet programme to other sites in Singapore.

In conclusion, this RCT will provide insights into a 6-month group exercise single intervention fall prevention programme on its effectiveness and cost utility in a community setting for a broad population of community-dwelling older adults. If proven effective, healthcare professionals, FIs, and community partners will be able to leverage on a community falls prevention programme that can be scaled nationwide.

## Supporting information

S1 Appendix SPIRIT 2013 checklist. (DOCX)

S2 Appendix Trial study protocol. (DOCX)
