# Supplementary material for: A community-based single fall prevention exercise intervention for older adults (STEADY FEET): Study protocol for a randomised controlled trial
Source: PLoS One. 2022 Oct 20;17(10):e0276385. doi: 10.1371/journal.pone.0276385 (PMC9584377; doi:10.1371/journal.pone.0276385)
Supplement: S2 Appendix — (DOCX) [file pone.0276385.s002.docx]

| **BACKGROUND AND RATIONALE** |
| --- |
| Falls in the elderly are a leading cause of death, disability, and health care costs.(1, 2) In community-dwelling older adults, nearly one-third experience at least one fall each year (3). About half of those with a previous fall will have a recurrent fall in the next year. (4, 5) As a result, about one-third of individuals who fall will seek medical attention and 10% will be hospitalized (6). In Singapore, the prevalence of falls among older adults aged 60 years and above is about 18.6%, while recurrent falls is about 6.4% (7). In Eastern Singapore, this would translate to about ~1,700 falls related admissions annually among 1/3 of fallers (51,360). About 50% of fallers (~ 25700) will be expected to fall again in the following year.  It is well established that poorer functional performance is associated with falls in older adults (8-10). Such adverse performance following falls has been shown to be modifiable by exercises. Exercise is shown to be an effective evidence-based intervention in improving functional performance. Systematic reviews have concluded that exercise programmes can reduce the rate of falls and risk of falling in community-dwelling older adults (11-13).  A 6-months community based structured exercise programme, Steady Feet (SF) aimed at improving physical strength and balance among older adults (aged 60 years and above) who are at high risk of falls was developed and implemented. Since its implementation, SF has been assessed to be feasible and has the potential to bring about improvements in its intended outcomes. Based on the learnings from first year implementation, refinements to the programme has also been made. As the next step, we seek to study the functional and clinical effectiveness of the programme and the effect of the programme on healthcare cost. |
| **HYPOTHESIS AND OBJECTIVES** |
| The primary objective of the Randomised Controlled Trial is to determine the effectiveness of a tailored structured community exercise programme in improving functional outcomes in older adults with a high risk of falls. The primary outcome of interest would be the difference in Short Performance Physical Battery (SPPB) scores between the intervention and control groups at 6 months. It is hypothesized that the intervention group will show ≥ 0.6 points improvements in SPPB scores compared to the control group.  Part 2 secondary objectives are to:   1. Evaluate the effect of the exercise programme on balance confidence 2. Evaluate the effect of the exercise programme on fear of falling 3. Evaluate the effect of the exercise programme on participants’ quality of life 4. Evaluate the effect of the exercise programme on falls rates 5. Evaluate the effect of the exercise programme on healthcare cost 6. Evaluate the adoption of the exercise programme 7. Understand the factors contributing to long-term sustainability of SF for further development and implementation 8. Evaluate the programme’s reach and fidelity |
|  |
| **EXPECTED RIKS AND BENEFITS** |
| There is a slight risk of injuries during the exercise classes. However, all necessary precautions will be taken to minimize this risk and all exercises are supervised by trained personnel. Potential benefits include improved strength, and balance. |
| **STUDY POPULATION** |
| List the number and nature of subjects to be enrolled. |
| The RCT will enrol 260 community-dwelling older adults aged 60 years and above who meet the study’s eligibility criteria, of which, a subset of them will be offered to participate in a qualitative interviews/FGDs to determine the implementation outcome of SF and to understand factors contributing to long-term sustainability of SF for further development and implementation. |
| Criteria for Recruitment and Recruitment Process |
| Initially, older adults will be screened for their risk of falls using the adapted Fall Risk for Older People – community setting (FROP-COM) screener, during community events. Individuals who are identified to be of a high risk of falls will then undergo a comprehensive geriatric assessment, vision test and cognitive tests, and screened to see if they meet the study’s eligibility criteria.  The study team will also reach out to community providers/instructors who are involved in SF. Those who meet the criteria will have an explanation of the study provided by a study team member and be invited to participate in the study. Only those who are agreeable to participate and able to provide informed consent will be recruited into the study. |
| Inclusion Criteria Community-dwelling participants will be eligible if they meet all the following inclusion criteria during screening:   1. Male or female aged 60 years and older 2. SPPB score: 7 – 10 3. Passes at least 2 out of 3 Vision Function Test(s) (LogMar vision, Stereoscopic vision, MET) 4. Does not possess significant cognitive impairment (AMT ≥ 5)   Community providers or instructors will be eligible if they meet all the following inclusion criteria:   1. Male or female aged 21 years and older 2. Are involved in the planning or implementation of the exercise programme |
| Exclusion Criteria |
| Community-dwelling participants will be excluded if they meet any of the following criteria during screening:   1. Male or female aged < 60 years old 2. SPPB score of ≥ 11 or ≤ 6 3. Did not pass at least 2 out of 3 Vision Function Test(s) (LogMar vision, Stereoscopic vision, MET) 4. Possess significant cognitive impairment (AMT < 5)   Community providers or instructors will be excluded if they meet all the following inclusion criteria:   1. Male or female below 21 years old 2. Not involved in the planning or implementation of the exercise programme |
|  |
| **STUDY DESIGN AND PROCEDURES/METHODOLOGY** |
| **Randomised controlled trial; community-dwelling older adults at high risks of falls**   - To determine the effects of SF on functional outcomes   Control (*n* = 130)   - Education and advice on how to reduce your risk of falls   Intervention (*n* = 130)   - 6-months exercise programme - Education and advice on how to reduce your risk of falls   A study comprising of (i) an open-label, parallel randomized controlled trial, and (ii) qualitative interviews or focus group discussions will be performed to assess the aims of the study. Crucial implementation outcomes, including reach, and fidelity, will also be collected.  Based on sample size calculations for the RCT (details can be found in Section 8), the study will involve 260 older adults aged 60 years and above who are at high risk of falls, and a subset will be recruited for the qualitative interviews/discussions.  After enrolment into the study, allocation concealment will be used to randomize participants to the intervention or control group before the baseline visit. Participants will be followed up prospectively for the next 6 months.  Participants will undergo the following activities.  **Intervention Group**:   - Attend 3 evaluation assessments during the study period. - Participate in 6 months of exercises   The exercises are divided into 2 phases:   - The Steady Feet (SF) exercise programme phase. A twice weekly tailored structured group exercise class that will be conducted for 3 months with community instructors and exercise video. - A 3 months maintenance exercise phase. A once weekly structured community group exercise class selected by participants from a recommended list of exercises [list is provided by the study team].   **Control Group**:   - Attend 3 evaluation assessments during the study period.   Primary Variable   - Functional assessment i.e. Short Physical Performance Battery (SPPB)   Secondary Variable(s)   - Balance confidence i.e. ConfBAL scale - Fear of Falling - Quality of life i.e. EQ-5D-5L - History of injurious falls - Single Leg Stance (SLS) - Four Square Step Test (FSST) - Timed Up and Go (TUG) - 30 second chair stand test (30CST) - 6-minute walk test (6MWT) - Healthcare utilisation - Cost of healthcare utilisation - Competency level of community instructors   Socio-demographic variable(s) and other supportive variables   - Age, gender, ethnicity, marital status, education level, residential type - Anthropometric measurement: weight and height - Measure of co-morbidities, i.e. Charlson Comorbidity Index (CCI) - Physical activity levels i.e. Global Physical Activity Questionnaire (GPAQ) - Satisfaction and open-ended feedback of community providers and instructors - Satisfaction and open-ended feedback of participants - Borg Rate of Perceived Exertion (RPE) - Attendance rate of exercise classes - Uptake rate of community-based exercises   **Study procedures and Visit Schedule**  Randomised controlled trial:  For intervention group:  Participants will take part in 6 months of community-based exercises that will commence within 2 weeks from the baseline visit.   \| Visit \| Visit 1  Baseline^1^  Day 1 \| Visit 2  3-month  Day 90 + 30 days^2^ \| Visit 3  6-month  Day 180 + 30 days \| \| --- \| --- \| --- \| --- \| \| Evaluation assessment visit procedures \| - Functional assessments - Questions on:   - Physical Activity levels   - Confidence balance   - Quality of Life   - History of injurious falls \| - Functional assessments - Questions on:   - Physical Activity levels   - Confidence balance   - Quality of Life   - Healthcare utilisation and related costs   - Programme satisfaction \| - Functional assessments - Questions on:   - Physical Activity levels   - Confidence balance   - Quality of Life   - History of injurious falls   - Healthcare utilisation and related costs   - Programme satisfaction \|   For controls:   \| Visit \| Visit 1  Baseline^1^  Day 1 \| Visit 2  3-month  Day 90 + 30 days \| Visit 3  6-month  Day 180 + 30 days \| \| --- \| --- \| --- \| --- \| \| Evaluation assessment visit procedures \| - Functional assessments - Questions on:   - Physical Activity levels   - Confidence balance   - Quality of Life   - History of injurious falls \| - Functional assessments - Questions on:   - Physical Activity levels   - Confidence balance   - Quality of Life   - Healthcare utilisation and related costs \| - Functional assessments - Questions on:   - Physical Activity levels   - Confidence balance   - Quality of Life   - History of injurious falls   - Healthcare utilisation and related costs \|   ^1^Baseline visit will take place within 50 days from the screening, and within 2 weeks prior to the commencement of the intervention group’s exercise classes.  ^2^Visit 2 will be conducted on a separate day after the last scheduled exercise class has been completed.  Participation in the RCT study is voluntary in nature. Participants may withdraw from participation in the study at any time by informing the study team members. However, data that have been collected until the time of participant’s withdrawal will be kept and analysed.  Qualitative interviews or focus group discussions:  Participation in the qualitative interviews/discussions is voluntary in nature, and the purpose of this portion is to better understand the factors contributing to long-term sustainability of Steady Feet for further development and implementation. The interviews or focus group discussions will be audio-recorded, and participants can request to remove what they mentioned during the sessions. The sessions are expected to last no longer than 2 hours. |
| **Schedule of Data Collection (RCT)**   \| Assessments \| Baseline  Day 1 \| 3-month  Day 90 + 30 days \| 6-month  Day 180 + 30 days \| \| --- \| --- \| --- \| --- \| \| Socio-demographic variables \| X \| - \| - \| \| CCI \| X \| - \| - \| \| SPPB \| X \| X \| X \| \| SLS \| X \| X \| X \| \| Four Square Step Test (FSST) \| X \| X \| X \| \| Timed Up and Go (TUG) \| X \| X \| X \| \| 30 second chair stand test (30CST) \| X \| X \| X \| \| 6-minute walk test (6MWT) \| X \| X \| X \| \| Borg Rate of Perceived Exertion (RPE) \| X \| X \| X \| \| Fear of Falling \| X \| X \| X \| \| ConfBAL scale \| X \| X \| X \| \| Global Physical Activity Questionnaire (GPAQ) \| X \| X \| X \| \| EQ-5D-5L \| X \| X \| X \| \| History of injurious falls \| X \| - \| X \| \| Participant satisfaction survey \| - \| X \| X \| \| Provider satisfaction survey \| - \| X \| - \| \| Healthcare utilisation \| - \| X \| X \| \| Cost of healthcare utilisation \| - \| X \| X \| \| Other implementation outcomes   - Enrolment rates - Retention rates - Completion rates - Adherence to programme protocol - Dosage of intervention \| X \| X \| X \|   Note:   - Baseline socio-demographic variables and CCI will be obtained from community screening and clinical records. - Age will be captured as participant’s age at the point of enrollment - Healthcare utilisation and cost would include ED visits, SOC visits, primary care visits, and hospital admission during the entire 6-month study period. Data might be extracted from clinical records. - Implementation outcomes such as enrolment, retention, dosage of intervention and completion rates will be obtained from administrative records that are captured routinely as part of the study. - Information on adherence to the programme protocol will be obtained from the same competency checklist administered to community instructors under the part 1 feasibility study. |
| **SAFETY MEASUREMENTS** |
| Definitions |
| Serious adverse event (SAE) in relation to human biomedical research, means any untoward medical occurrence as a result of any human biomedical research which:   - results in or contributes to death - is life-threatening - requires in-patient hospitalisation or prolongation of existing hospitalisation - results in or contributes to persistent or significant disability/incapacity or - results in or contributes to a congenital anomaly/birth defect - results in such other events as may be prescribed   Adverse event (AE) in relation to human biomedical research means any untoward medical occurrence as a result of any human biomedical research which is NOT serious. Adverse event can be any unfavourable and unintended sign (including an abnormal laboratory finding), symptom, or disease possibly/ probably/ definitely associated with the participant in the human biomedical research. |
| Collecting, Recording and Reporting of Serious Adverse Events (SAEs) to CIRB |
| Only related SAEs (definitely/ probably/ possibly) will be reported to CIRB. Related means there is a reasonable possibility that the event may have been caused by participation in the research. Please refer to the CIRB website for more information on Reporting Requirement and Timeline for Serious Adverse Events.  The investigator is responsible for informing CIRB after first knowledge that the case qualifies for reporting. Follow-up information will be actively sought and submitted as it becomes available.  Related AEs will not be reported to CIRB. However, the investigator is responsible to keep record of such AEs cases at the Study Site File. |
| Safety Monitoring Plan |
| The study team will conduct, at least, quarterly meeting to review the study data. Study data to be reviewed will include compilation of data obtained from study and any adverse events and/or serious adverse events recorded to ensure that patient safety is adhered. |
| Complaint Handling |
| Any participant complains will be raised to the PI and dealt with on a case-by-case basis. |
| **DATA ANALYSIS** |
| Data Quality Assurance |
| Data will be routinely reviewed for the purposes of tracking the progress of the study, assuring accuracy, and completeness of the data. Adverse events or serious adverse events will be recorded and monitored to ensure the safety of the participants. |
| Data Entry and Storage |
| Data will be collected via 1) hard copy data collection forms and/or 2) extracted electronically from hospital clinical systems, and/or 3) digital audio recorders. Data will be entered into an electronic database, and together with audio recordings will be stored on a password protected laptop accessible only to the PI, Co-Investigators and study team members. |
| **SAMPLE SIZE AND STATISTICAL METHODS** |
| Determination of Sample Size |
| The primary outcome for the study is the difference in SPPB scores between intervention and control group at 6 months.  We estimated a predicted effect size of around 0.4, at 80% power using a two-sided 0.05 level t-test. Accounting for an attrition rate of around 25%, an estimated sample size of 130 participants per group was obtained. Sample size calculations were calculated with G*Power (ver. 3.1.9.4).  An adaptive design may be used after the interim analysis. Sample size re-estimation may be performed to evaluate the power to detect a statistically significant difference in primary outcome between the intervention group and the control group based on an interim analysis. |
| Statistical and Analytical Plans **Statistical and Analytical Plans**  Data Sets to Be Analyzed  The primary analysis will include all available data from all participants who were randomized into the intervention or control groups. This is the intention to treat data set.  Analysis of Variable(s)  RCT analysis will accord with the “intention-to-treat” principle. A *p* < 0.05 will be considered as statistically significant.  Categorical data will be presented as frequency (percentage), while continuous data will be presented as mean (standard deviation) for parametric distribution and median (interquartile range) for non-parametric distribution. Group or pre/post comparisons of categorical and continuous variables will be performed using chi-square test/ Fisher’s Exact test and/or ANOVA as appropriate.  Categorical variables might be examined using logistics regression models. Demographics and baseline variables will be examined between groups and might be used for adjustment of the regression models. The between group differences in primary and secondary outcomes will be examined using regression and/or ANOVA models.  Cost analysis will examine total healthcare cost from primary care to acute hospital stays during the study period.  If data transformations (e.g., logarithmic) are used to improve distribution characteristics (e.g., normality, variance homogeneity) for parametric procedures, they will be described in reporting of statistical results.  Audio recordings will be transcribed verbatim, translated if needed, and then analysed using content or thematic analysis.  Interim Analysis Plan  An interim analysis might be conducted when approximately 70 participants per group have a known 6^th^ month primary outcome.  Criteria for Early Termination of the Trial  After the interim analysis, sample size and study power might be re-estimated. There are no plans to terminate the trial early. |
|  |
| **DIRECT ACCESS TO SOURCE DATA/DOCUMENTS** |
| The investigator(s)/institution(s) will permit study-related monitoring, audits and/or IRB review and regulatory inspection(s), providing direct access to source data/document. |
|  |
| **QUALITY CONTROL AND QUALITY ASSURANCE** |
| Study related personnel will be trained by the study team on the protocol, proper use of data collection form, informed consent procedures, randomization, maintenance of essential study documents and any other study procedures such as study assessments, before study initiation.  The study-trained investigators or study coordinator may conduct any additional training of study centre personnel after study initiation.  **Study Monitoring**  The study monitor will schedule planned monitoring visits throughout the study to ensure that:   1. Rights and well-being of human participants are protected; 2. Study protocol is being followed; 3. Accurate, complete and current source documents are being maintained; 4. Reports are being made to the IRB; 5. Conduct of the study is in accordance with ICH, GCP and applicable regulatory guidance; 6. Data collection forms and all electronic data capture are a complete and accurate representation of the source documents and; 7. A screening log of all participants considered for enrollment is maintained. |
|  |
| **ETHICAL CONSIDERATIONS** |
| This study will be conducted in accordance with the ethical principles that have their origin in the Declaration of Helsinki and that are consistent with the Good Clinical Practice and the applicable regulatory requirements.  This final Study Protocol, including the final version of the Participant Information and Consent Form, must be approved in writing by the Centralised Institutional Review Board (CIRB), prior to enrolment of any patient into the study.  The principle investigator is responsible for informing the CIRB of any amendments to the protocol or other study-related documents, as per local requirement. |
| Informed Consent |
| If an individual is deemed to be eligible, a designated study team member for consent taking will provide the participant with the Informed Consent Form and Patient Information Sheet. the designated team member will countersign on the Form once the participant has consented and this process would be done in the presence of a witness. For participants who are unable to sign, thumbprint may be used for these cases and done in the presence of a witness. |
| Confidentiality of Data and Patient Records |
| Research data will be stored in a password protected laptop within the site. Screening forms, source documents and signed informed consent forms will be stored in a locked cupboard only accessible by the PI, Co-investigators or study team members. |
| **PUBLICATIONS** |
| The team understands that the information from this study will be used for the development of a community based structured exercise programme, and therefore, may be disclosed, at the discretion of the PI to other government agencies or approved partners.  The Changi General Hospital team will be involved in the preparation of any proposed publications of study findings, including abstracts. Individuals from our partner organisations may be invited to be an author or contributor to a study publication. |
|  |
| **RETENTION OF STUDY DOCUMENTS** |
| Research data for all participants will be retained in a secured storage facility for a minimum of 7 years after completion of research study or date of publication of the research using the research data, whichever is later. |
| **FUNDING and INSURANCE** |
| This study is funded by the Ministry of Health (MOH) Falls Prevention under the National Innovation Challenge (NIC) for Active and Confident Ageing Grant.  **References**  1. Tinetti ME, Kumar C. The patient who falls: "It's always a trade-off". Jama. 2010;303(3):258-66.  2. Tinetti ME. Where is the vision for fall prevention? J Am Geriatr Soc. 2001;49(5):676-7.  3. Tromp A, Pluijm S, Smit J, Deeg D, Bouter L, P.M L. Fall-risk screening test: A prospective study on predictors for falls in community-dwelling elderly. Journal of clinical epidemiology. 2001;54:837-44.  4. Tinetti ME, Speechley M. Prevention of Falls among the Elderly. New England Journal of Medicine. 1989;320(16):1055-9.  5. Lim SC. Elderly Fallers: What Do We Need to Do? Proceedings of Singapore Healthcare. 2010;19(2):154-8.  6. Nevitt MC, Cummings SR, Kidd S, Black D. Risk factors for recurrent nonsyncopal falls. A prospective study. Jama. 1989;261(18):2663-8.  7. Dai W, Tham Y-C, Chee M-L, Tan NYQ, Wong K-H, Majithia S, et al. Falls and Recurrent Falls among Adults in A Multi-ethnic Asian Population: The Singapore Epidemiology of Eye Diseases Study. Scientific Reports. 2018;8(1):7575.  8. Lauretani F, Ticinesi A, Gionti L, Prati B, Nouvenne A, Tana C, et al. Short-Physical Performance Battery (SPPB) score is associated with falls in older outpatients. Aging Clin Exp Res. 2019;31(10):1435-42.  9. Hars M, Audet M-C, Herrmann F, De Chassey J, Rizzoli R, Reny J-L, et al. Functional Performances on Admission Predict In-Hospital Falls, Injurious Falls, and Fractures in Older Patients: A Prospective Study. Journal of Bone and Mineral Research. 2018;33(5):852-9.  10. Veronese N, Bolzetta F, Toffanello ED, Zambon S, De Rui M, Perissinotto E, et al. Association between Short Physical Performance Battery and falls in older people: the Progetto Veneto Anziani Study. Rejuvenation research. 2014;17(3):276-84.  11. Sherrington C, Tiedemann A, Fairhall N, Close JC, Lord SR. Exercise to prevent falls in older adults: an updated meta-analysis and best practice recommendations. N S W Public Health Bull. 2011;22(3-4):78-83.  12. Gillespie LD, Robertson MC, Gillespie WJ, Sherrington C, Gates S, Clemson LM, et al. Interventions for preventing falls in older people living in the community. Cochrane Database Syst Rev. 2012(9):Cd007146.  13. Matchar DB, Duncan PW, Lien CT, Ong MEH, Lee M, Gao F, et al. Randomized Controlled Trial of Screening, Risk Modification, and Physical Therapy to Prevent Falls Among the Elderly Recently Discharged From the Emergency Department to the Community: The Steps to Avoid Falls in the Elderly Study. Arch Phys Med Rehabil. 2017;98(6):1086-96. |
